# Three-Dimensional Printing of Fetal Models of Congenital Heart Disease Derived From Microfocus Computed Tomography: A Case Series

**DOI:** 10.3389/fped.2019.00567

**Published:** 2020-01-21

**Authors:** Camilla Sandrini, Claudio Lombardi, Andrew I. U. Shearn, Maria Victoria Ordonez, Massimo Caputo, Francesca Presti, Giovanni Battista Luciani, Lucia Rossetti, Giovanni Biglino

**Affiliations:** ^1^Division of Cardiology, Department of Medicine, University of Verona, Verona, Italy; ^2^Studio Diagnostico Eco, Vimercate, Italy; ^3^Bristol Medical School, Bristol Heart Institute, University of Bristol, Bristol, United Kingdom; ^4^Division of Obstetrics and Gynecology B, Department of Surgery, Dentistry, Pediatrics and Gynecology, University of Verona, Verona, Italy; ^5^Division of Cardiac Surgery, Department of Surgery, Dentistry, Pediatrics and Gynecology, University of Verona, Verona, Italy; ^6^National Heart and Lung Institute, Imperial College London, London, United Kingdom

**Keywords:** 3D printing, microfocus computed tomography, fetal heart, congenital heart disease, fetal cardiology, prenatal diagnosis

## Abstract

This article presents a case series of *n* = 21 models of fetal cardiovascular anatomies obtained from post mortem microfocus computed tomography (micro-CT) data. The case series includes a broad range of diagnoses (e.g., tetralogy of Fallot, hypoplastic left heart syndrome, dextrocardia, double outlet right ventricle, atrio-ventricular septal defect) and cases also had a range of associated extra-cardiac malformations (e.g., VACTERL syndrome, central nervous system anomalies, renal anomalies). All cases were successfully reconstructed from the microfocus computed tomography data, demonstrating the feasibility of the technique and of the protocols, including in-house printing with a desktop 3D printer (Form2, Formlabs). All models were printed in 1:1 scale as well as with the 5-fold magnification, to provide insight into the intra-cardiac structures. Possible uses of the models include education and training.

## Introduction

Congenital heart defects are the most frequent congenital malformations. Advances in prenatal medicine allow early diagnosis, starting from the eleventh week of gestation (1, 2). Confirming the cardiac malformation after termination of pregnancy is essential for medical knowledge and familial counseling. Conventional autopsy is the gold standard technique for post mortem confirmation of congenital heart disease (CHD) but it lacks diagnostic power especially in challenging samples, such as cases of termination of pregnancy and of low dimension/weight (3, 4). In recent years, radiologic tools are becoming valid alternatives to conventional autopsies, in light of their non-destructive approach and high-resolution imaging capabilities (5–12). By using X-ray source on contrast-enhanced samples, post mortem microfocus computed tomography (micro-CT) can reach a spatial resolution of voxel sizes below 1 micron (i.e., <0.001 mm), thus allowing the evaluation of cardiac structures also in challenging specimens (5–9). Iodine preparation of the sample is necessary in order to create contrast between different cardiac soft tissues, that are otherwise not distinguishable (6).

Three-dimensional (3D) printing in CHD is gaining increasing interest in the clinical and bioengineering communities alike, considering the breath of its applications. These include medical education, surgical, and catheter-based procedural individualized planning, and manufacturing research for device innovation (13–16). At present, applications of 3D printing in fetal cardiology are limited, yet the technique is appealing as precise visualization of small structures is essential to achieve a correct diagnosis, even in a very early in pregnancy. Pioneering work coming from *in utero* 3D echocardiography/magnetic resonance imaging (MRI) (17, 18) and from post mortem micro-CT (19) have been recently published.

Based on these early experiences, here we present a feasibility study of 3D printing normal fetal hearts and fetal hearts with CHD from micro-CT datasets, resulting in a case series of different diagnoses. The focus of the article is on technical issues of the imaging and printing processes.

## Materials and Methods

### Selection of Cases

Post mortem micro-CT followed by conventional autopsy is offered to all patients referred to our Center for fetal echocardiography who decided for termination of pregnancy (below 21+6 week of gestation, according to Italian Law) for either fetal cardiac anomalies or extra-cardiac malformations. Samples are managed according to previous described procedure (20). Post mortem micro-CT acquisitions are performed using a micro-CT SkyScan 1176 scanner (Bruker, Kontich, Belgio). Here we present the experience of consecutive cases collected between July 2016 and December 2018.

### Micro-CT Imaging Protocol

Before performing micro-CT scans, the specimens were stained by immersion in Lugol solution 15% (10 g potassium iodide and 5 g iodine in 100 ml water) for 24 h. Subsequently, the heart was washed with alcohol to remove free iodine, blotted dry, and scanned All scans were performed with a Bruker Skyscan 1176 micro-CT scanner (Bruker, Brussels Kontich, Belgium) with a gantry diameter of 68 mm. Fixed acquisition times of 25 min were used for resolutions at 9 μm and 6 min for resolution at 18 μm. The exposure time was 1,050 ms, respectively, the voltage was 80 kV in all cases, the current was 300 μA, and the rotation step 0.30°. The projection data were corrected for distortion and reconstructed by adjusting, smoothing and correction of ring artifact and beam hardening. The reconstructed isotropic voxel size was 9. Images were reconstructed with the scanner software (NRecon 1.6.6.0, Skyscan, Brucker micro-CT, Belgium) and converted to DICOM format for analysis. The time for reconstruction ranged from 2 to 60 min, depending on the volume of the sample. Acquired images were rendered and exported as a three-dimensional (3D) volume. Post-processing analysis was performed using CTVox volume rendering 64 bit version, DATAVIEWER 64 bit version (Bruker, Kontich, Belgium), and Horos (v 2.4) software (free and open source code software at Horosproject.org).

### 3D Printing Protocol

The micro-CT data served as the input for the 3D printing process. Images were imported in commercial software (Mimics v.21, Materialize, Leuven, Belgium) and reconstructed following steps of thresholding, region growing and semi-automatic image segmentation with manual editing where required, and the final volume meshes were exported as.stl files. All models were 1:1 in size as well as scaled with a 5-fold factor to provide more insight such small-sized cardiovascular structures. Each case was thus printed twice, in the two sizes. Prior to printing, models' surfaces were checked, and a scaffold was added (PreForm, Formlabs, …). Models were printed in-house using a rigid white resin (Form2, Formlabs) and cleaned manually through standard steps of isopropanol washing and curing with final manual removal of the model's scaffold.

## Results

A total of *n* = 21 cases were collected and reconstructed. Anamnestic data are summarized in Table 1. Samples consisted in heart (19/21, 90%) and heart-lungs (2/21, 10%). Mean gestational age at fetal echocardiography was 15.0 ± 2.9 weeks (range: 12–21 weeks). Mean gestational age at termination of pregnancy was 16.0 ± 3.0 weeks (range: 12–22 weeks). Overall, 18/21 (86%) cases suffered from cardiac anomalies, 15 (83%) of which were complex CHD. 13/21 (62%) cases had abnormal karyotype at invasive testing (chorionic villus sampling or amniocentesis). 10/21 (48%) cases had associated extra-cardiac malformations (VACTERL syndrome, central nervous system anomalies including omphalocele and other anomalies of the posterior cranial fossa, cystic hygroma, cleft lips, renal anomalies including ptosis and polycystic kidneys, skeletal anomalies including anomalies of the arms, legs, vertebral column, and ribcage). Mean longitudinal diameter was 1.05 ± 0.37 cm (range 0.4–1.7 cm). Mean transverse diameter was 0.95 ± 0.36 cm (range 0.4–1.8 cm). Mean weight was 0.88 ± 1.06 g (range: 0.39–4 g). Table 2 summarizes micro-CT acquisition properties.

**Table 1:** Summary of cases included for 3D modeling, including main diagnosis, gestational age (GA) at diagnosis, and termination of pregnancy (TOP), karyotype, extra-cardiac anomalies, and dimensional assessment.

	**Prenatal data**					**Macroscopic data**			
**ID**	**GA at diagnosis (week+days)**	**Diagnosis**	**Kariotype**	**Extra-cardiac anomalies**	**GA at top (week+days)**	**Sample**	**Longitudinal diameter (cm)**	**Transversal diameter (cm)**	**Weight pre micro-CT (g)**
1	20	Posterior malalignment VSD, aortic hypoplasia, hypoplastic aortic arch	46, XX	VACTERL syndrome	20+4	Heart	1.50	1.30	3.370
2	16+2	TOF	46, XX	Frontal bossing, hands anomalies	18+6	Heart	1.50	1.30	3.070
3	20+2	Large ASD, large VSD, borderline LV, aortic hypoplasia	46, XY, del (11)(q23.3)	Labial cleft, kidney, and hands anomalies	20+4	Heart	1.50	1.20	3.330
4	16+1	VSD	47, XY, +21	–	16+5	Heart	1.00	0.80	1.320
5	21+2	HLHS (mitral atresia/aortic atresia)	–	–	21+6	Heart	1.70	1.80	4.000
6	16+2	PA-VSD/truncus arteriosus	47, XY, +9	IUGR, CNS anomalies	16+3	Heart	1.10	0.80	0.650
7	12+4	AVSD	47, XY, +21	–	13+6	Heart	0.60	0.50	0.390
8	14+3	AVSD/large VSD inlet	47, XY, +21	–	14+3	Heart	1.00	0.70	0.490
9	17+2	TOF	–	Omphalocele	18+2	Heart	1.40	1.30	1.800
10	13+2	AVSD	47, XY, +21	–	13+4	Heart	0.50	0.50	0.400
11	14	Dextrocardia, large conoventricular VSD, probable DORV, side-by-side great arteries	47, XXX	CNS and kidney anomalies	17+1	Heart	0.80	0.80	0.687
12	17+4	Normal	46, XX	Fetal hydrops, pleural, and abdominal effusion	17+5	Heart	0.90	0.90	0.641
13	13+3	Normal	–	–	16+0	Heart	1.00	1.00	0.821
14	12+5	AVSD/ partial AVSD (inlet VSD+little ostium primum ASD)	47, XY, +21	–	12+5	Heart	0.40	0.40	0.687
15	12+5	AVSD	47, XX, +21	–	15	Heart	1.20	1.20	0.641
16	16+4	Muscular VSD, right aortic arch	47, XX, +21	–	17+5	Heart + Lungs	1.50	1.50	0.821
17	19	Normal	–	Fetal hydrops, cystic hygroma, bilateral pleural effusion	12+6	Heart	0.80	0.80	0.641
18	20	AVSD	47, XY, +21	Acrania	14+6	Heart + Lungs	1.10	1.10	0.821
19	21	AVSD	47, XX, +21	–	12+6	Heart			0.687
20	22	TOF/anterior malalignment VSD	47, XX, +21	–	13+3	Heart			0.641
21	23	Inlet VSD	–	–	21+3	Heart			0.821

**Table 2:** Main settings for micro-CT acquisitions.

**ID**	**Top to micro-CT (days)**	**Lugol %**	**Lugol (h)**	**Filter**	**Resolution (μm)**	**Energy range (V)**	**Current range (μA)**	**Rotational step (^**°**^)**
1	68	25	72	Cu+Al	18	500	89	264
2	194	25	72	Cu+Al	18	500	89	264
3	260	25	72	Cu+Al	18	500	89	264
4	178	20	72	Cu+Al	18	500	89	264
5	21	25	72	Cu+Al	18	500	89	264
6	18	20	72	Al 0,5 mm	18	210	50	500
7	16	20	72	Al 0,5 mm	9	900	50	500
8	16	20	72	Al 0,5 mm	18	210	50	500
9	17	25	72	Cu+Al	18	500	89	264
10	16	20	72	Al 0,5 mm	9	900	50	500
11	247	20	72	Cu+Al	18	300	80	300
12	200	20	72	Cu+Al	18	300	80	300
13	200	20	72	Cu+Al	18	300	80	300
14	83	15	48	Al 0,5 mm	18	210	50	500
15	80	30	72	Cu+Al	18	300	80	300
16	74	30	72	Cu+Al	18	300	80	300
17	67	20	48	Al 0,5 mm	18	210	50	500
18	66	30	72	Cu+Al	18	300	80	300
19	33	15	48	Al 0,5 mm	18	210	50	500
20	41	30	72	Cu+Al	18	300	80	300
21	19	25	72	Cu+Al	18	300	80	300

**Figure 1:**
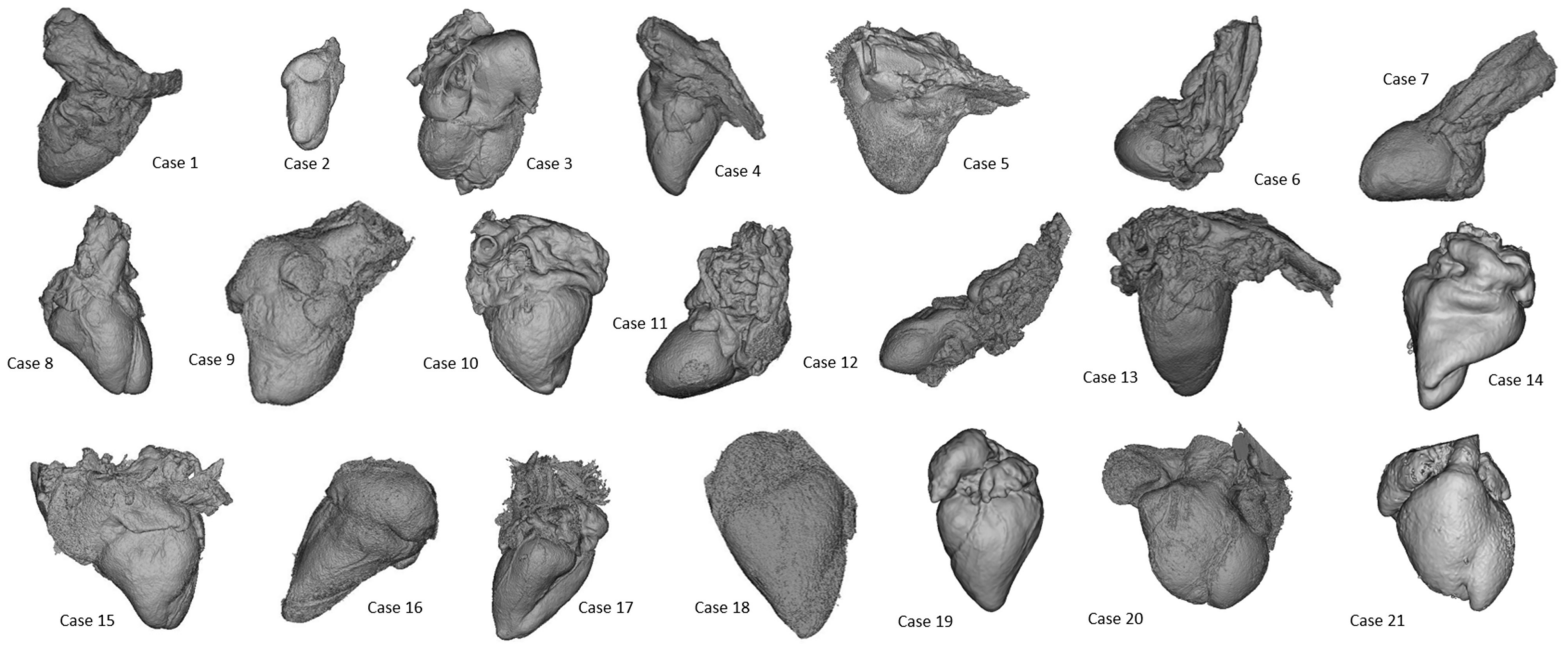
Presentation of 3D models of all patients included in the case series, highlighting the wide range of morphologies (not to scale).

**Figure 2:**
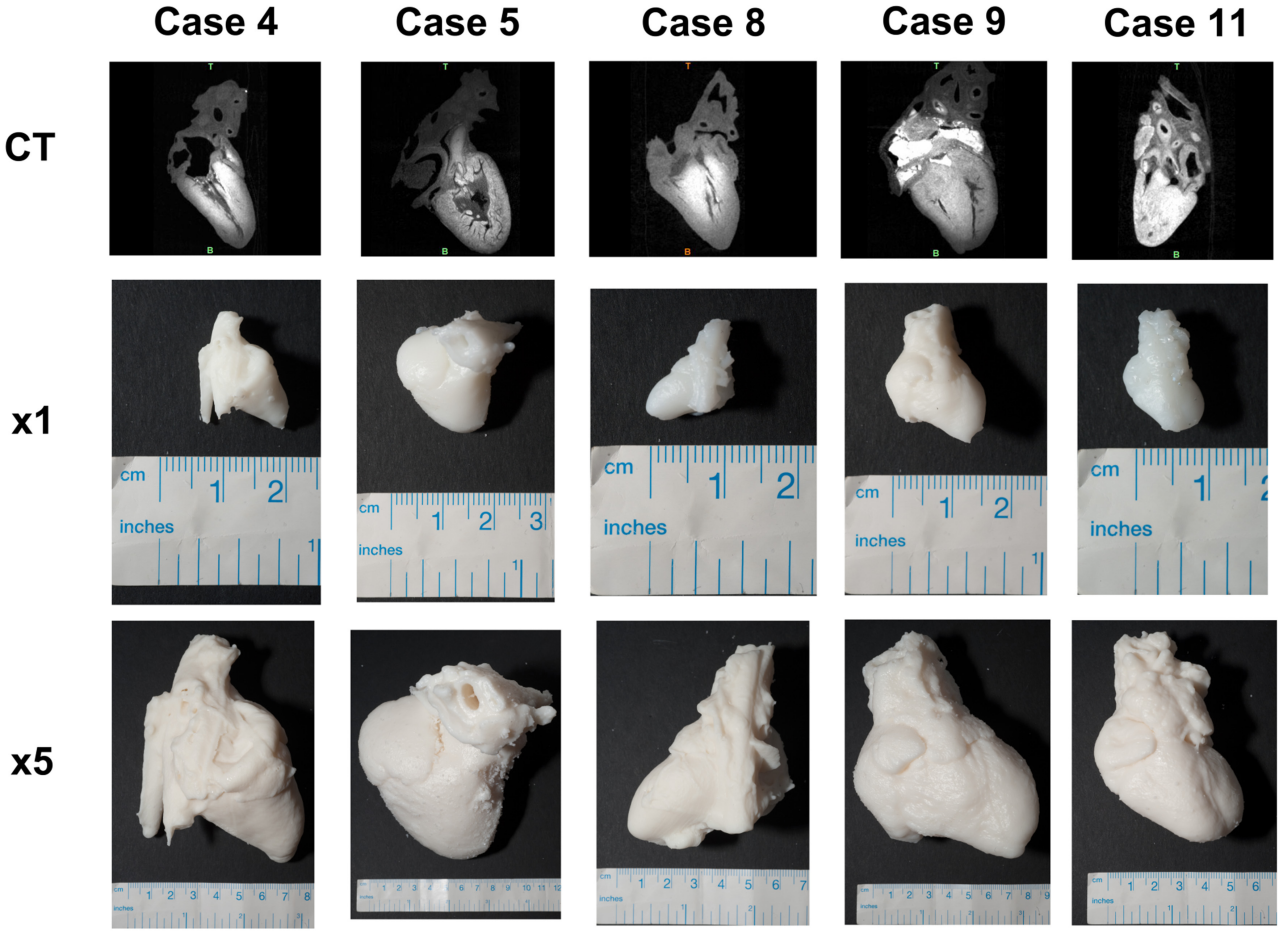
Example of 3D printed models for a range of cases with different diagnoses, including the 1:1 3D printed model as well as the corresponding 5-fold scaled model for each case.

All cases were successfully reconstructed, demonstrating the feasibility of the technique and of the protocols, including in-house printing. Examples of 3D reconstructions are provided in Figure 1. Examples of 3D printed models are provided in Figure 2. When printed with the 5-fold magnification, models were also cut in a four-chamber view equivalent section, to provide insight into the intra-cardiac structures.

## Discussion and Conclusion

Little is known about the feasibility of 3D printing from micro-CT datasets in the field of fetal cardiology. Traditionally, 3D printing of cardiovascular models relies on cardiac CT and MRI datasets of pediatric and adult human hearts in different settings according to established methodologies (12). Accuracy of postmortem micro-CT in *ex-vivo* evaluation of human fetal heart is validated. More recently, proof of principle experiences in 3D printing from micro-CT of human specimens in craniofacial surgery, placental imaging, archeological remains, bone imaging, and cardiac anatomy has been presented (19). Shelmerdine et al. reported one example of 3D printing from micro-CT of an human fetal heart of 16 weeks of gestation, demonstrating that both main cardiac segments (atria, ventricles, and great vessels) and smaller component (cardiac valves, branching of the pulmonary artery and aorta, papillary muscle, and ventricular trabeculations) can be identified (19). There are any published data concerning earlier gestational age and larger case series. Our study complements these information on the feasibility of the technique and presents a case series comprehensive of complex cases of CHD, including hypoplastic left heart syndrome, double outlet right ventricle and tetralogy of Fallot from early and mid-gestational age (from 12 to 21 weeks of gestation). We agree with Shelmerdine's experience on feasibility and possible utility of 3D printing of human fetal heart from micro-CT. As compared to previous published projects, the novelty we would like to demonstrate concerns the feasibility of micro-CT in a larger population of normal and pathological hearts with a wide range of gestational ages. The studied population consists of both normal and abnormal fetal hearts. Whilst the segmentation and printing processes are the same for normal and pathologic specimens, reconstruction of complex structures may require expert guidance in the identification of anatomical features of interest. Moreover, the population includes small samples (15/21 cases, 71%: weight ≤ 1 g; 9/21 cases, 43%: longitudinal and transverse diameter ≤ 1 cm) and cases of low gestational age (10/21 cases, 48%: termination of pregnancy before the 15 weeks of gestation). This enabled us to demonstrate the feasibility of the process for both larger and smaller samples.

We focused only on technical aspects. Educational, research, and clinical implications remain to be addressed in the future and are beyond the scope of presenting this case series.

Virtual and printed models are nowadays used for didactic and research purposes in other areas of medicine, e.g., 3D printing fetal brains (22). These models can potentially represent a valuable resource for teaching fetal cardiac anatomy to medical students and trainees as well, presenting both normal fetal cardiovascular anatomy and congenital heart defects of different complexity. Whilst this should be systematically assessed in the context of cardiac morphology teaching, evidence is beginning to suggest that accurate 3D printed replicas can improve the teaching experience, reducing potential adverse reactions from students (e.g., in relation to cultural or religious backgrounds) and also holding the potential to show fetal development at different stages, to illustrate its full progression (21). Educational values of 3D printing models from micro-CT could play even a more important role in our era, where early fetal echocardiography is offered to high risk pregnancies since the eleventh week of gestation. The possibility to visualize small cardiac structure and to navigate into normal and pathological fetal heart offered by micro-CT could facilitate interpretation of echo images and, ultimately, improve medical knowledge. Moreover, integration of different imaging modalities also opens the door to research applications, which include 3D printing fetal heart models indeed derived from fetal echocardiographic data and integrated with 4D MRI flow information, which can also represent a valuable platform for *in vitro* modeling (18). Future application of 3D printed models could be related to familial counseling, fetal diagnosis, and more individualized planning of fetal and neonatal therapeutic strategies. On one hand, counseling applications may be limited due to the potentially confrontational and highly sensitive nature of the conversations and the role of the model as a communication tool in this context may potentially be counterproductive, as “reopening the wound” in revisiting the clinical conversations that ultimately led to terminating the pregnancy. On the other hand, 3D models (even scaled-up) could be used during familial counseling to facilitate familial awareness of fetal cardiac dimension and anatomy. Though biological effect of X ray radiation limits the *in-vivo* use of micro-CT and avoid patient-specific models, 3D reconstruction of first/early second trimester cardiac structure from micro-CT datasets is feasible. On the contrary, at present, data from 3D fetal echocardiography acquired during the first trimester has never been used to reconstruct and print fetal heart.

## Data Availability Statement

The datasets generated for this study are available on request to the corresponding author.

## Ethics Statement

Ethical review and approval was not required for the study on human participants in accordance with the local legislation and institutional requirements. The patients/participants provided their written informed consent to participate in this study. Written informed consent was obtained from the individual(s) for the publication of any potentially identifiable images or data included in this article.

## Author Contributions

CS: conception and design of study, analysis and interpretation of data, and drafting of manuscript. CL: conception and design of study, drafting of manuscript, and final approval. AS and MO: analysis and interpretation of data. MC, FP, GL, and LR: final approval. GB: conception and design of study, analysis and interpretation of data, drafting of manuscript, and final approval.

### Conflict of Interest

The authors declare that the research was conducted in the absence of any commercial or financial relationships that could be construed as a potential conflict of interest.
